# Alternative Method for Determination of Vibroacoustic Material Parameters for Building Applications

**DOI:** 10.3390/ma17123042

**Published:** 2024-06-20

**Authors:** Krzysztof Nering, Konrad Nering

**Affiliations:** 1 Faculty of Civil Engineering, Cracow University of Technology, 31-155 Cracow, Poland; 2 Faculty of Mechanical Engineering, Cracow University of Technology, 31-155 Cracow, Poland; konrad.nering@pk.edu.pl

**Keywords:** acoustic comfort, vibrational comfort, material properties, damping, dynamic stiffness, polyurethane, rubber granulate, image processing, rebound, indentation

## Abstract

The development of urbanization and the resulting expansion of residential and transport infrastructures pose new challenges related to ensuring comfort for city dwellers. The emission of transport vibrations and household noise reduces the quality of life in the city. To counteract this unfavorable phenomenon, vibration isolation is widely used to reduce the propagation of vibrations and noise. A proper selection of vibration isolation is necessary to ensure comfort. This selection can be made based on a deep understanding of the material parameters of the vibration isolation used. This mainly includes dynamic stiffness and damping. This article presents a comparison of the method for testing dynamic stiffness and damping using a single degree of freedom (SDOF) system and the method using image processing, which involves tracking the movement of a free-falling steel ball onto a sample of the tested material. Rubber granules, rubber granules with rubber fibers, and rebound polyurethanes were selected for testing. Strong correlations were found between the relative indentation and dynamic stiffness (at 10–60 MN/m^3^) and the relative rebound and damping (for 6–12%). Additionally, a very strong relationship was determined between the density and fraction of the critical damping factor/dynamic stiffness. The relative indentation and relative rebound measurement methods can be used as an alternative method to measure the dynamic stiffness and critical damping factor, respectively.

## 1. Introduction

More and more people live in cities, and this trend only seems likely to continue [1,2,3,4,5,6]. The growth of the urban population causes the development of urbanization and, consequently, the transport infrastructure, which must meet increasingly greater challenges [7,8,9,10,11]. Urban planners make compromises related to the proximity of residential buildings to the railway and road infrastructure. This translates into shortening the travel time of city residents, which is undoubtedly a desirable phenomenon [12,13,14]. However, the price for such a development is the increased presence of bothersome stimuli, such as noise and vibrations, especially from the railway infrastructure [15,16,17,18,19,20,21,22].

The development of urbanization also imposes the need to increase population density, which is reflected not only in the way buildings are located, but also in the increase in the number of inhabitants in a single building [23,24,25,26,27]. This is a necessary response to the market situation [28,29,30], but the consequence is also a reduction in the comfort of residents by increasing the noise from neighbors to which residents are exposed [28,31,32,33].

The expansion of transport infrastructure and housing construction, undoubtedly beneficial to national economies, reduces the quality of life. Therefore, it is necessary to consciously design the infrastructure, taking into account the nuisance of its use. A reduction in harmful stimuli such as noise and vibration is required to ensure the health and well-being of urban inhabitants. The answer to the above challenges is the introduction of appropriate directives, regulations [34,35], and standards [36,37,38,39,40,41,42] that constitute the means and the goal to be achieved as well as the methodology for achieving this goal. The constantly developed approach to assessing comfort requires a good understanding of how humans perceive bothersome stimuli [21,43,44,45,46,47]. Ways to ensure comfort require a lot of information about the technology and materials used [48,49].

There are various ways to reduce the emission of harmful stimuli [50,51,52,53,54,55]. It is worth mentioning the use of vibration isolation on railway tracks, which allows for the reduction in the propagation of vibrations generated during the passage of a train [56,57,58]. This has a positive impact not only on buildings located in the vibration influence zone [59,60,61,62,63], but also on residents who are exposed to a smaller dose of annoying vibrations [64,65,66,67].

In residential and office buildings, i.e., places intended for people to stay over long periods at a time, noise protection is used in the form of floating floors or raised flooring [68,69,70,71,72]. Partitions are selected appropriately so that they increase acoustic insulation. This allows for the reduction in exposure to airborne and impact noise [73,74,75,76].

Also, with the increasing popularity of mechanical ventilation, which is dictated by energy efficiency [77,78,79], it is worth mentioning the use of passive vibration isolation of technical devices and duct silencers in order to reduce noise emissions related to the operation of ventilation system devices [80,81,82,83,84].

Vibration isolation also plays an important role in controlling seismic actions on the building. A proper assessment of vibration isolation parameters such as stiffness and damping is key to predict a building’s response to seismic actions [85,86,87]. An appropriate design of the structure allows for the control of resonance frequencies in the context of seismic forces and appropriate control over the internal forces in the structure [88,89,90].

The dominant material in passive vibration isolation used in various construction sectors is rubber [91,92,93,94,95]. This material has very good features related to stiffness, dimensional stability, and damping. Materials derived from waste are also used, such as rubber granules [96,97,98] or polyurethanes [99,100,101,102]. The fact that polyurethanes can be recycled materials increases their potential in terms of sustainable development. In residential construction, elastified polystyrene and mineral wool dominate as vibration-insulating materials for floors [103,104,105,106,107], but there is a potential to use rebound polyurethanes because they have appropriate stiffness parameters.

The most important material parameters from the point of view of vibration isolation are dynamic stiffness and damping [108,109,110,111,112,113,114,115]. These parameters allow for the adequate design of a passive vibration isolation system. This allows the designer to properly assess if a given material is suitable for the added solution. For residential construction, the material assessment method is determined according to the ISO 9052-1 standard [48], where the tests allow for the estimation of the dynamic stiffness under small stresses (2 kPa). Additionally, the second parameter, namely, the damping of the material, can also be assessed; this will have a significant impact when designing an experiment, considering the impact of vibrations on people [100,115,116].

The aim of this article is to validate an alternative method for estimating the value of dynamic stiffness at low stresses [48] and damping [100] using image analysis during a free ball drop. This study is intended to allow for a faster assessment of these parameters. The usefulness of the alternative method can be indicated especially during preliminary research—for the purpose of the preliminary selection of materials or estimation of the above-mentioned parameters for research purposes.

## 2. Measurement Methodology and Setup

The selected materials for the research were vibration-isolating materials: rebound polyurethane, rubber granules, and rubber granules with rubber fibers. The dimensions of the samples were 200 mm × 200 mm × 50 mm. The compilation of material parameters is presented in Table 1.

**Figure 1:**
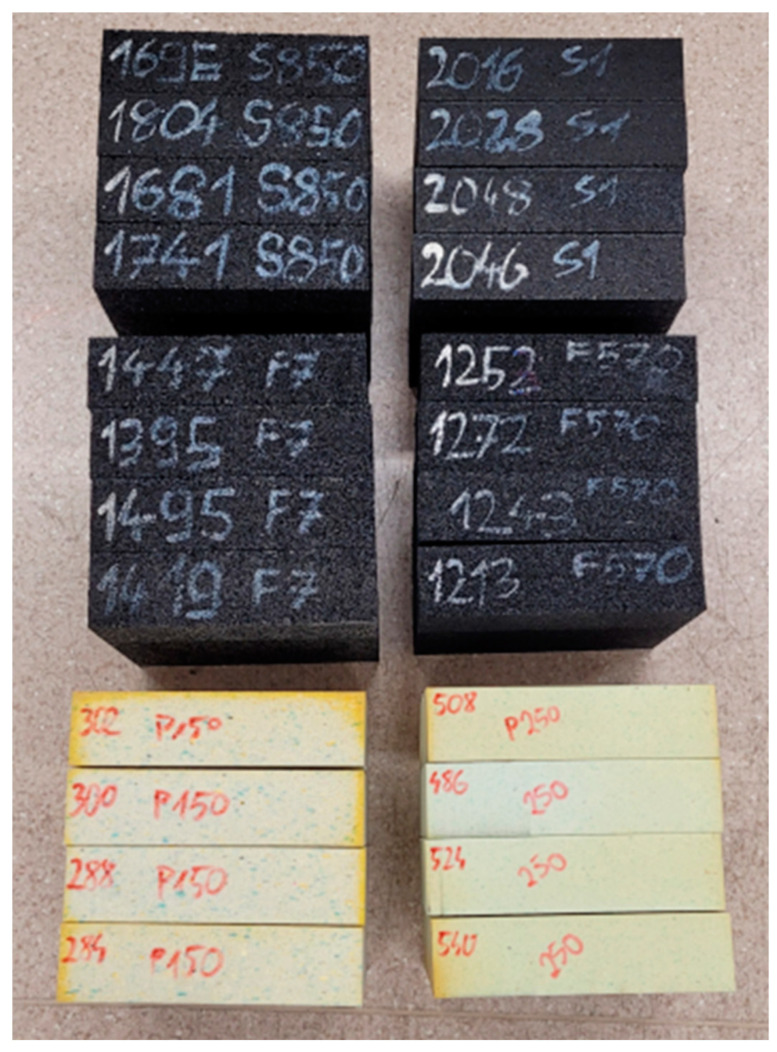
Samples used for research in this article. Tested materials are rubber granulate (S), rubber granulate with rubber fibers (F), and rebound polyurethane (P).

The samples tested in this article are presented in Figure 1.

The choice of materials was dictated by several factors. First of all, the selected materials were to be used as passive vibration isolation. Another factor was the widest possible variation in the density of the materials. The fact that the materials had to differ in stiffness and compressibility was also taken into account.

### 2.1. Dynamic Stiffness and Critical Damping Factor Estimation with Reference Method

In this paper, measurements were made using a dynamic stiffness testing machine [48,100], which determined the dynamic stiffness of the material and the critical damping fraction.

**Figure 2:**
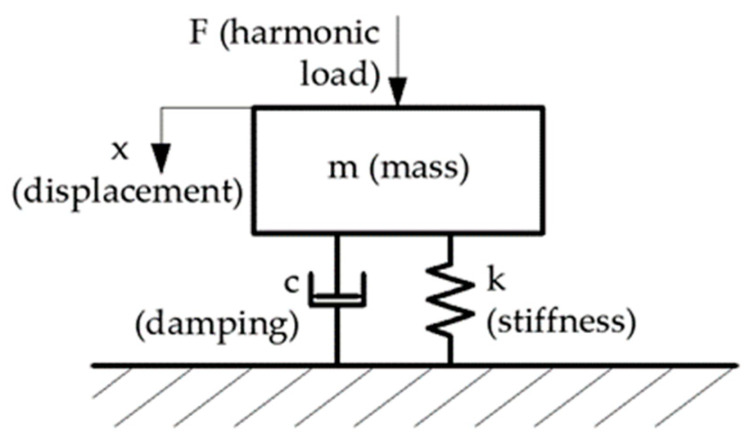
Physical model of the mass–damper–spring system with single degree of freedom (SDOF).

This method involved determining the dynamic response of the system, which was modelled using a single degree of freedom (SDOF) system as shown in Figure 2. Based on the dynamic response, the resonant frequency and the critical damping fraction were determined.

The resonance frequency was estimated by locating the maximum value in the vibration acceleration response spectrum from the frequency domain analysis of the response. Then, the resonant frequency was converted using the formula for the dynamic stiffness of the frame with Equation (1).

(1) $$DS=4\pi^{2}m\prime f_{r}^{2}$$

where *DS* is dynamic stiffness, *m*′ is total mass per unit area used during the test, and *f_r_* is resonant frequency

**Figure 3:**
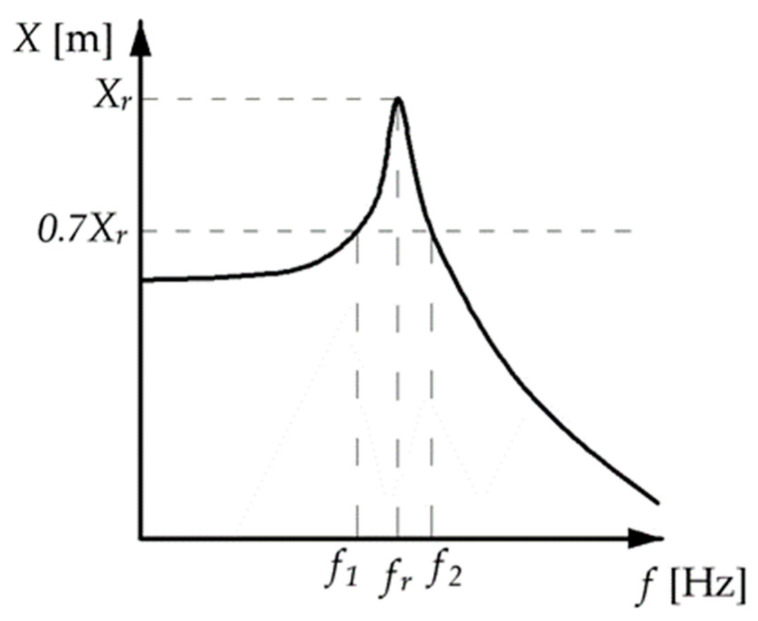
Schematic presentation of the half-power bandwidth method using the displacement spectrum. *X_r_*—displacement amplitude, *f_r_*—resonant frequency, and *f*_1_ and *f*_2_—correspond to frequencies to 0.7 value of resonance amplitude.

The critical damping fraction was estimated using the half-power method. The scheme for determining the critical damping fraction is shown in Figure 3.

Once obtaining the values according to the above scheme, the damping relationships were described mathematically using Equation (2) [100,117,118].

(2) $$\frac{f_{2}-f_{1}}{f_{r}}=\frac{\delta }{\pi \sqrt{1-{\left(\frac{\delta }{2\pi }\right)}^{2}}}$$

Equation (3) describes the relationship by which the critical damping fraction was determined [19,100].

(3) $$\delta =\frac{2\pi D}{\sqrt{1-D^{2}}}$$

The description of the parameters of the machine used for dynamic stiffness testing is presented in Table 2.

**Figure 4:**
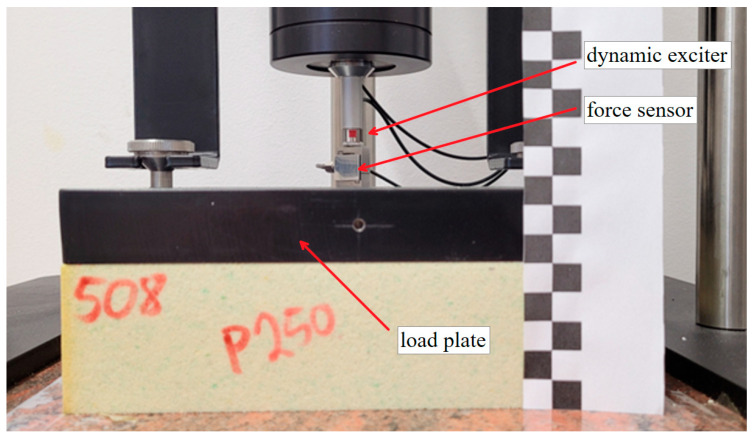
Dynamic stiffness test bench with loaded sample. One square in photograph is one centimeter.

The machine depicted in Figure 4 was utilized for conducting tests on vibroacoustic parameters. Primarily designed for assessing dynamic stiffness, this machine incorporates a dynamic exciter, which applies harmonic force to the load plate via a force sensor. The load plate, weighing 8 kg, is positioned atop the test sample with a pre-load static force ranging from 0.1 to 0.4 N. The system’s response is measured using an IEPE (Integrated Electronics Piezo-Electric) accelerometer.

**Figure 5:**
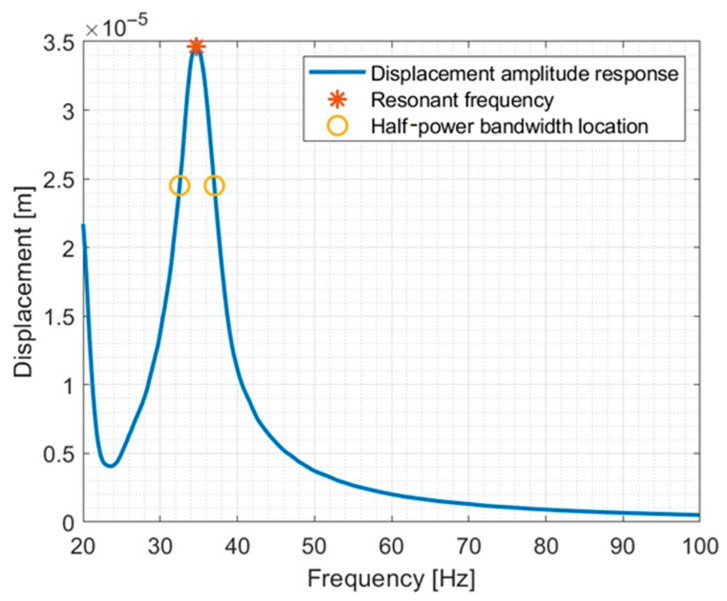
Pseudo-displacement response spectrum of sample P150 (288 kg/m^3^) with resonant frequency of 35.2 Hz and critical damping factor 0.0645.

The testing system was dynamically excited using a sinusoidal force generated by the exciter. The amplitude of the applied sinusoidal force was maintained at 0.4 N with a tolerance of ±0.005 N. The frequency range spanned from 1 Hz to 100 Hz. The frequency was incremented by 0.1 Hz every second during the measurement period. The system response was registered using an IEPE accelerometer positioned at the load plate. Exemplary result is shown in Figure 5.

### 2.2. Alteranative Method

The image processing method was used as an alternative method proposed in this paper. This method involves tracking the movement of a steel ball in free fall from a given height and observing its behavior during and after rebound from the tested material. This method determines local extremes in the time course of the ball’s position.

A high-speed camera was used to record the position of the ball. In order to identify local maxima, the optical axis of the camera lens was set halfway between the starting (drop) point and the upper surface of the sample. To record local minima, the optical axis of the camera lens was set in the plane of the upper surface of the sample.

**Figure 6:**
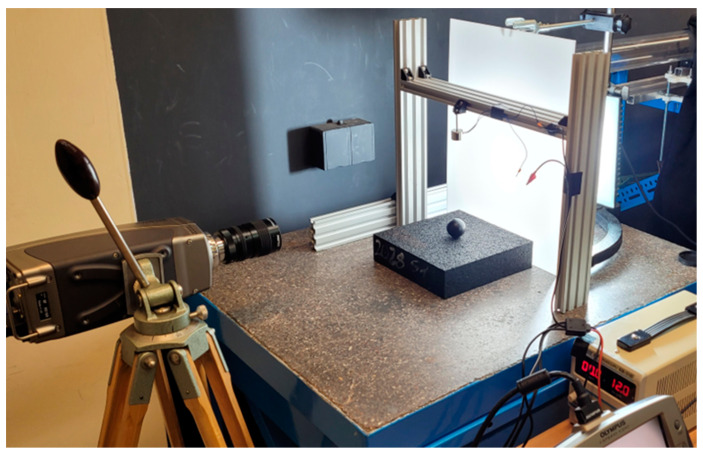
Test bench for ball-tracking experiment.

**Figure 7:**
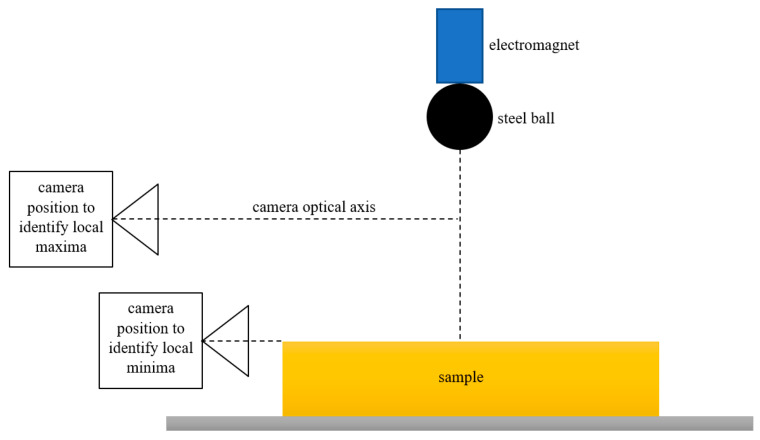
Test bench diagram.

In the research using this method, an Olympus (Tokyo, Japan) I-Speed high-speed camera with a frame rate of 1000 fps was used. An electromagnet released by a toggle switch was used to free drop the steel ball. In order to ensure good contrast to the falling ball, the scene was illuminated using a high-performance nonflickering LED reflector. To standardize the lighting, a diffuser made of white matt plexiglass was used. The ball was dropped into the center of the sample surface in order to uniform the stress distribution in the sample. The measurement setup is shown in Figure 6. The diagram is shown in Figure 7.

Steel balls (with diameter of 34 mm and mass of 160.5 g) were used for the test. A steel ball was chosen due to the significant difference in stiffness between the tested material and steel. In order to reduce reflections on the ball, which would introduce contamination to the image (and the position of the ball), the ball was painted with matte black paint.

The height from which the ball was freely dropped was 180 mm. This height was chosen due to the capabilities of the camera lens and the ability to measurably observe the ball throughout its entire course. Another argument for this drop height was the possibility of recording the ball’s indentation in the material (local minimum of the ball’s position). In the case of preliminary tests at lower drop heights, there were problems with registering the local minimum. At higher altitudes, the speed of the ball falling right after the collision was so high that the error in determining the position of the ball at a given number of frames of the recorded image increased significantly.

The choice of two camera positions—close and far (15 cm and 40 cm away from the center of the ball, respectively)—was dictated by two requirements. The first one was to enable the recording of the first reflections and the beginning of the drop. The second was a more accurate recording of the indentation. Although the far position allowed us to achieve these two requirements with one camera position, a close position was also used to increase the accuracy of the indentation analysis.

Six ball drops were performed for one sample. This decision was made to increase the robustness of the results by determining the average ball path. After each drop, it was verified if any local yielding occurred at the point of contact between the sample and the ball. There were no permanent deformations of the tested materials.

**Figure 8:**
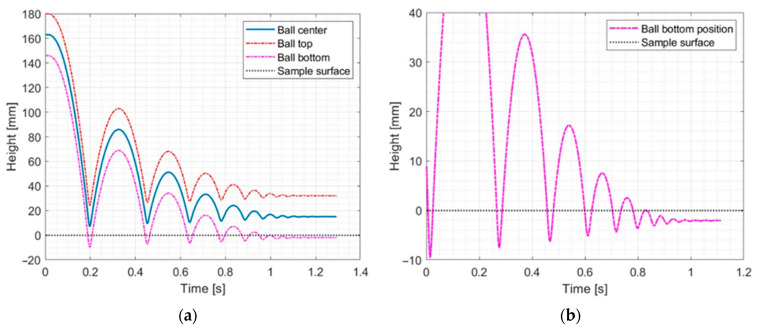
Examples of ball-tracking results for the P150 sample with a density of 288 kg/m^3^. (**a**) shows full motion of the ball, and (**b**) shows magnification in ball drop area.

As the last step, indentation was calculated based on ball tracking. Indentation was defined as the maximum absolute value of the ball bottom position during contact with sample (local minimum values are presented in Figure 8). Indentation was determined for each of the observed contacts. The rebound height was determined as local maximum values of ball tracking (with ball bottom position).

## 3. Results

The results for the reference method are presented in Table 3. The presented results are a summary of the averaged values of the critical damping factor (CDF), resonant frequency (Rf), and dynamic stiffness (DS) for each of the tested materials. Additionally, the values for the 95% confidence intervals (95%CI) are presented in parentheses.

The post-processed and averaged results of tracing the ball during free fall are presented in Table 4 and Table 5. Table 4 shows the results of the relative bounce height up to the 4th bounce. The relative bounce height was calculated in the following manner: The first relative rebound height was the ratio of the start height to the first rebound height (1st peak/start). The second relative rebound height was the ratio of the second to the first rebound height (2nd/1st peak). The third relative rebound height was the ratio of the third to the second rebound height (3rd/2nd peak). And the last and fourth rebound height was calculated in the same manner. Additionally, the values for the 95% confidence intervals (95%CI) of the calculated relative rebound heights are presented in parentheses.

Table 5 shows the results for the averaged and post-processed relative indentations. Similar to the results with the relative rebound height, it was decided to show the results only up to the 4th indentation. The relative indentation was calculated in the following manner: The first relative indentation (1st ind.*/start) was the ratio of the first indentation depth to the start position of the ball bottom. The second relative indentation (2nd/1st ind.) was the ratio of the second indentation depth to the first rebound height with the ball bottom position. The third and forth relative indentations were calculated in the same way. Additionally, the values for the 95% confidence intervals (95%CI) of the calculated relative indentations are presented in parentheses.

In the next part of the paper, an examination of any relationships between the reference method for testing dynamic stiffness and the relative values measured by ball tracking was performed.

## 4. Discussion

First of all, the question must be raised as to if the alternative method can be related to the reference method in any way. The reference method has its basis in the literature [100,115] and ISO standard [48].

### 4.1. Dependencies between Relative Indentation and Dynamic Stiffness

As a first topic, it can be discussed if the indentation can provide direct information about the stiffness of the material. The indentation itself strongly depends on the height of the drop; hence, the decision was made to use relative indentation, which shows the ratio of the rebound height of subsequent indices. While examining various mathematical models of the relationship between the relative indentation and dynamic stiffness, it was decided to choose a linear model using a linear function. Goodness of fit is presented in Table 6.

**Figure 9:**
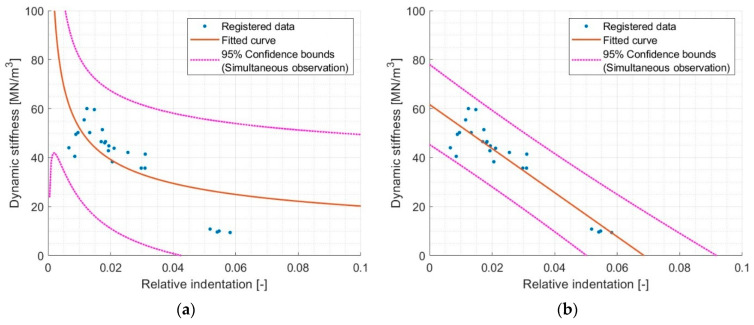
Comparison of different models of dynamic stiffness and relative indentation relationship. (**a**) model Power1 val(x) = a·x^b^, where a = 7.834, b = −0.4115, and R^2^ = 0.5435 and (**b**) model Linear val(x) = p1·x + p2, where p1 = −899.6, p2 = 61.65, and R^2^ = 0.8587.

From the data included in Table 6 it can be concluded that the first relative indentation to dynamic stiffness correlates best. However, this raises some doubts, because a linear function has a zero crossing (unless it is constantly positive) and also accepts negative arguments (which would mean that relative indentation can be negative). For this reason, it is worth considering another function describing the relationship between relative indentation and dynamic stiffness. As an alternative to the linear function, one can propose the rational function or power function, which is characterized by asymptotic behavior. The analyses show a better R^2^ for the power function, which was (however) still not satisfactory (R^2^~0.5). The matching results are shown in Figure 9.

Although the power function has a physical justification—it does not allow negative dynamic stiffness or relative indentation—the R^2^ for the linear function is much higher (R^2^~0.54 to R^2^~0.86, respectively). It can therefore be said that in a limited range of relative indentation (0.01 to 0.06), fitting with a linear function gives sufficient accuracy.

Focusing on the results, it can be noticed that in the relative indentation near value 0.01, there is a large dispersion of the results for dynamic stiffness. These are the results for samples S1000 (lowest relative indentation) and S850 (relative indentation ~0.015). There may be several reasons for this condition. The first reason is the apparent softening of the S1000 samples due to a lack of full contact with the pressure plate due to their granulation. The second reason may be limitations related to the accuracy of recording small indentations. Although the mentioned reasons constituting limitations of the methods used are not the subject of this paper, it is worth taking a closer look at this problem in order to draw conclusions for further studies.

**Figure 10:**
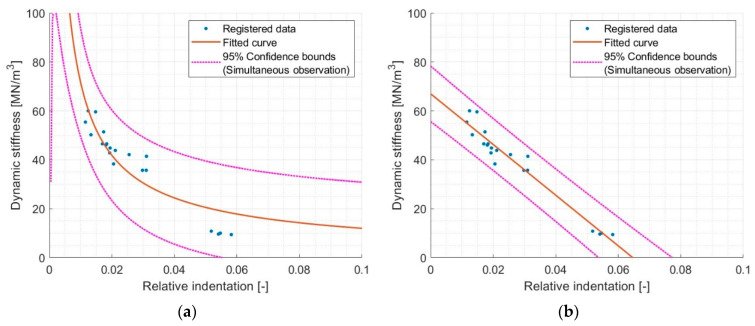
Comparison of different models of dynamic stiffness and relative indentation relationship with exclusion of S1000 (relative indentation < 0.01). (**a**) model Power1 val(x) = a·x^b^, where a = 2.000, b = −0.777, and R^2^ = 0.8381 and (**b**) model Linear val(x) = p1·x + p2, where p1 = −1036, p2 = 66.95, and R^2^ = 0.9468.

Therefore, it was decided to remove the S1000 samples from the analysis and repeat the previously presented analysis. The results are shown in Figure 10.

After removing the S1000 samples from the analysis, a significant improvement in correlation was obtained for both models. For the power function, an increase in R^2^ was obtained from ~0.55 to ~0.85, which allowed us to demonstrate an acceptable correlation. For the linear function, an increase in R^2^ was obtained from ~0.85 to ~0.95, which indicates a very good correlation.

In conclusion, it can be indicated that it is possible to determine dynamic stiffness by examining relative indentation. However, the obtained results indicate that this possibility exists to a limited extent in the relative indentation (0.01–0.06) for the tested sample geometry. Attempts to use possible extrapolation using the power and linear functions do not provide appropriate results.

### 4.2. Dependencies between Relative Rebound and Critical Damping Factor

Damping is the second parameter examined in this paper. In the case of damping, it is expected that the relative height to which the ball bounces will be correlated with the critical damping factor. This is due to the dissipation of the total energy of the ball by the sample during the rebound process.

As in the case of relative indentation, various models were tested for the relationship between the critical damping factor and the relative rebound. Yet again, it was noticed that the linear function gives the highest R^2^ for the recorded measurements. The results of this correlation for the subsequent relative rebound are presented in Table 7.

In the case of the first and second relative rebounds, a high R^2^ (~0.8) was observed, while in further relative rebounds, a significant decrease in the R^2^ value was observed.

Relative rebound, similar to relative indentation, correlates well with the linear function. However, this does not change the fact that there is no physical justification for such a model. Hence, there is a need to propose another, more rational function. It is known that in the theoretical case, when the material has no damping, that the ball will bounce endlessly. Naturally, this was assuming no air resistance and no damping in the ball itself. Hence, at a relative rebound equal to 1, the CDF should be equal to 0. Moreover, when increasing the CDF for the sample, the relative rebound decreases. In theory, the material may have damping above the critical damping, but in practice, such materials are not used.

**Figure 11:**
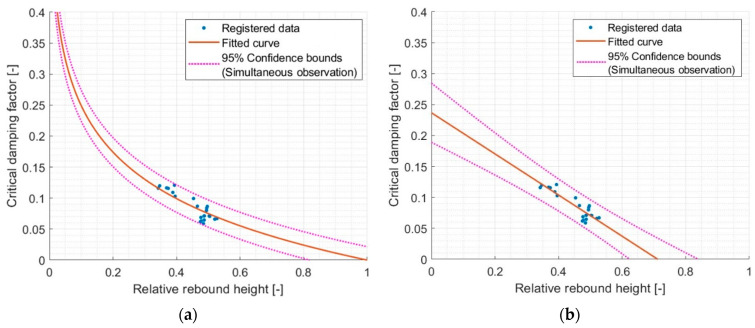
Comparison of different models of critical damping factor and first relative rebound relationship. (**a**) model Logarithmic val(x) = a·log10(x), where a = −0.2492 and R^2^ = 0.7654 and (**b**) model Linear val(x) = p1·x + p2, where p1 = −0.3325, p2 = 0.2368, and R^2^ = 0.8149.

Therefore, two functions that can describe the dependencies between the CDF and the relative rebound have been proposed—linear and logarithmic functions. The match results for the first relative rebound are shown in Figure 11.

In the case of a logarithmic function that preserves the boundary condition associated with the lack of damping, R^2^~0.77 was demonstrated, which means an acceptable correlation. For the best-fitting linear function, R^2^ is ~0.81, which already means a good correlation. However, the R^2^ values are comparable with a slight advantage of the linear function.

**Figure 12:**
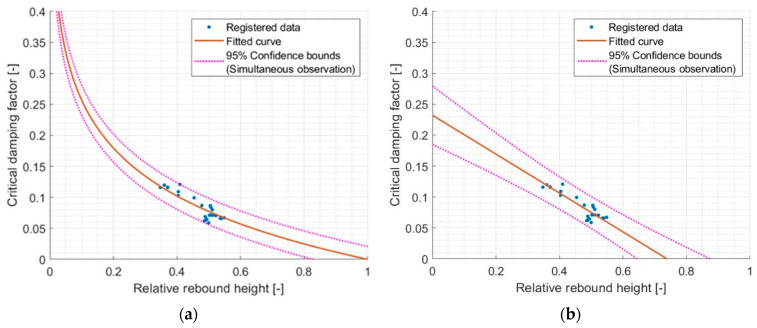
Comparison of different models of critical damping factor and first and second relative rebound (averaged) relationship. (**a**) model Logarithmic val(x) = a·log10(x), where a = −0.2568 and R^2^ = 0.7809 and (**b**) model Linear val(x) = p1·x + p2, where p1 = −0.3143, p2 = 0.2321, and R^2^ = 0.8123.

Taking into account the fact that for the results presented in the Table 7, the R^2^ was ~0.8 for the 1st and 2nd relative rebounds, it was decided to average the results for these two relative rebounds and make the same adjustment again. The results are shown in Figure 12.

For the logarithmic function, an increase in R^2^ was observed from 0.765 to 0.781. For the linear function, there was a slight decrease in R^2^ from 0.815 to 0.812. Therefore, it can be said that both models correctly predict the behavior of the dependence of the CDF on the relative rebound. It is worth mentioning that the model using the logarithmic function allows for some extrapolation of the results.

### 4.3. Reference Method—Density, Dynamic Stiffness, and Critical Damping Factor

Using the results from the reference method for analysis, we checked if there were any relationships between the measured values for the tested samples. Considering that the tests were carried out using materials with different internal structures, and for which the only common feature was their use as passive vibration isolation, having such trivial data as density, it would be beneficial to estimate their other mechanical parameters. However, strong correlations should not be expected, because material density is one of many parameters responsible for stiffness or damping.

**Figure 13:**
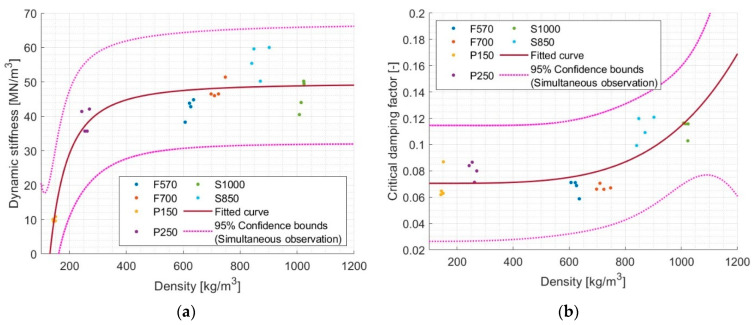
Models of Power2 (val(x) = a·x^b^ + c) describing relationship between (**a**) dynamic stiffness and density where a = −1.422 × 10^6^, b = −2.105, c = 49.53, and R^2^ = 0.8842 and (**b**) critical damping factor and density where a = 2.201 × 10^−15^, b = 4.433, c = 0.07053, and R^2^ = 0.6301.

Various models were tested to examine the dependence of dynamic stiffness on density and the critical damping factor on density. The highest R^2^ in both cases was obtained for the power function summed with the free term. Figure 13 shows the results of the discussed adjustments.

For the tested set of samples, describing the relationship between dynamic stiffness and density gives an R^2^ of 0.884. Although this would indicate a good correlation, it is difficult to speak of a more general principle. Taking into account the 95% confidence bounds, it turns out that according to the proposed model, for a density of, e.g., 800 kg/m^3^, the dynamic stiffness results are between ~30 MN/m^3^ and ~65 MN/m^3^. Consequently, even for engineering applications, this is a rather rough estimate of dynamic stiffness. The reason for this arrangement can be seen in the fact that rubbers dominate in high densities and rebound polyurethanes in low densities. On the other hand, we have the problematic S1000 material, which appears to have lower dynamic stiffness compared to less dense materials of a similar type.

With the dependence of the CDF on density, an R^2^ of 0.630 was obtained. Here, R^2^ shows a reduced ability to make the CDF predictions based on density. Of course, the problems indicated above could be discussed, but the graph shows that the attenuation increases significantly only at higher densities. When the large scatter at the 95% confidence bounds is ignored, it can be concluded that the attenuation increases with the density of the material.

**Figure 14:**
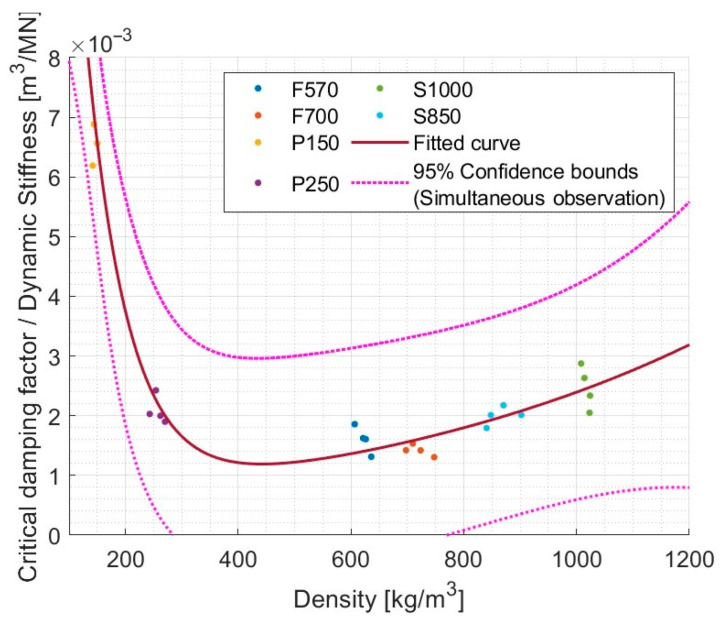
The fraction Critical damping factor/Dynamic stiffness as a function of density described with the usage of Exp2 model (val(x) = a·exp(b·x) + c·exp(d·x)), a = 0.04442, b = −0.01349, c = 0.0005713, d = 0.001433, and R^2^ = 0.9460.

Bearing in mind that in the tested materials the increase in dynamic stiffness with density occurs rather at the beginning of the analyzed density and the damping increases in the second half of this range, an attempt can be made to find a resultant relationship. It was checked if the CDF/DS ratio depends to some extent on density. The proposed model was a linear combination of two exponential functions. The result is presented in Figure 14.

The value of R^2^ = 0.946 indicates a very good correlation. For rubber materials, regardless of their internal structure—whether it is just granules or granules with fibers—a constant increase is observed. For rebound polyurethane, the analyzed value quickly decreases depending on the density. This allowed us to conclude that although there is no direct relationship between the dynamic stiffness or the CDF and density, there is an indication that the ratio of CDF/DS may depend directly on the density even for different materials. It is definitely worth further study.

### 4.4. Reference Method—Rayleigh Damping

**Figure 15:**
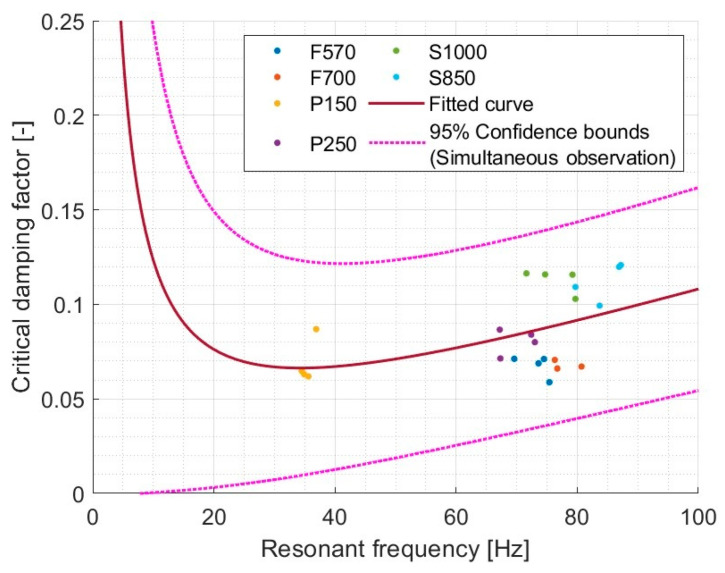
Rayleigh damping model (val(x) = 1/2·(a·x + b/x)), where a = 0.001935, b = 2.273, and R^2^ = 0.2385.

Rayleigh damping is widely used in computational methods [119,120,121] due to its simplicity and computational efficiency. Based on data from various materials, it was decided to check if Rayleigh damping can be considered on a dynamic stiffness testing machine. The results are shown in Figure 15.

In the case of the tested machine, for various materials subjected to the test procedure, no significant relationship was found that would indicate that there is Rayleigh damping in the form described in its definition, because R^2^ = 0.239 indicates the lack of a relationship.

**Figure 16:**
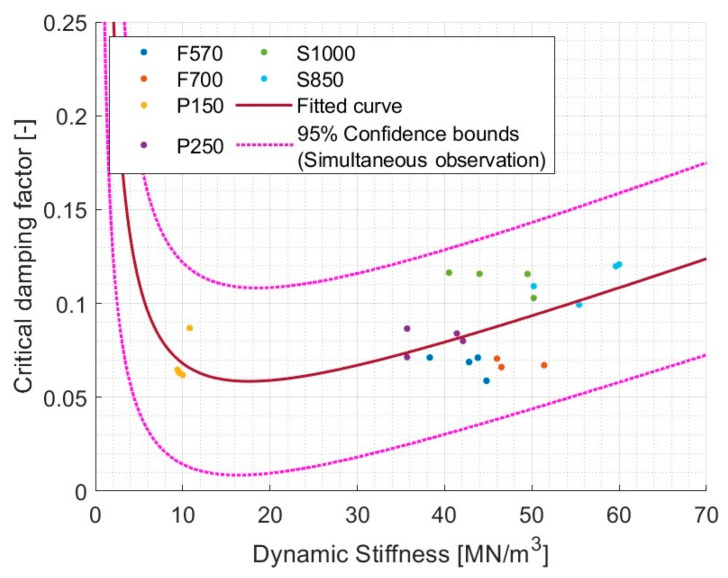
Rayleigh-ish damping model based on dynamic stiffness instead of resonant frequency (val(x) = 1/2·(a·x + b/x)), where a = 0.003327, b = 1.029, and R^2^ = 0.3730.

As an alternative to pure Rayleigh damping, it was checked if Rayleigh’s relationship between the CDF and the DS could be used. The results of this adjustment are shown in Figure 16.

Although a slightly higher R^2^ = 0.373 was obtained, it indicated a lack of a relationship in this case as well.

## 5. Conclusions

### General Conclusions and Further Studies

The following conclusions can be drawn from the research conducted for this paper.

The method using relative rebound and relative indentation gives a good prediction of the dynamic stiffness and critical damping factor parameters. Taking into account that the R^2^ in all models discussed is above 0.7, and in the case of certain limitations even above 0.9, it confirms that the alternative method of using image analysis is an effective estimate of material parameters.

It turns out that only the initial measurement (1st or eventually 2nd rebound) can be used for research. Moreover, when examining the relationship, it was in this region that the highest R^2^ values were obtained, which allows for the additional shortening of the test time without losing its accuracy. Very weak dependencies of dynamic stiffness on density and the critical damping factor on density were found. This makes it impossible to predict the above-mentioned parameters based on density. However, a certain trend can be identified where dynamic stiffness and damping increase with density.

An interesting observation is the strong relationship between the critical damping factor/dynamic stiffness fraction and material density. The R^2^ value is very high (~0.95). It can be concluded that for low densities, the damping is very effective per unit of dynamic stiffness. This efficiency drops to its minimum at approximately 400 kg/m^3^ and then gradually increases.

There is no strong relationship in the case of tests on a machine compliant with ISO 9052 [48] that would allow us to identify Rayleigh damping. In the case of the model using dynamic stiffness instead of resonant frequency, a certain trend can be noticed, showing an increase in the critical damping factor with the increase in dynamic stiffness.

The main disadvantage of an assessment using image processing is its accuracy. Although R^2^ values around 0.8 for the critical damping factor prediction and R^2^ values around 0.9 were obtained for the tested samples, the extrapolation of these results beyond the framework specified in the article for dynamic stiffness (at 10–60 MN/m^3^) and damping (for 6–12%) is problematic. Particular attention should be focused on the S1000 sample, which, although slightly lower in dynamic stiffness, showed lower relative indentation than the proposed models would indicate. It is worth emphasizing that the surface structure of the sample has a significant impact on the relative indentation. Samples with a large structure (large pore size on the surface) may appear softer when tested because the ball sinks deeper during contact.

The observed discrepancies related to the decrease in stiffness with increasing density for the S1000 material prompted an in-depth examination of the causes of this phenomenon. It is suspected that this may be related to the reduction in the contact surface of the pressure plate of the dynamic stiffness testing machine. The reason may also be found in the technology of making granules and the possibility to use a more resilient glue or reducing its amount compared to other granulates. Another way of distributing the granules can also be indicated. At this time, the cause of this condition is not clear; therefore, additional tests should be performed, taking into account the above considerations.

It is worth mentioning that ball tracking was performed using an algorithm that directly tracks the ball’s path. However, it is worth considering much more complex methods. One such method is Eulerian video magnification [122,123], which could allow for more accurate measurements, especially of the contact phenomenon during the free fall of the ball.

## Figures and Tables

**Table 1:** List of materials used for research.

Material Name	Sample ID	Density (kg/m^3^)	Density Averaged (kg/m^3^)
rubber granulate (S1000)	S1000_01	1008.0	1017.25
	S1000_02	1014.0	
	S1000_03	1023.0	
	S1000_04	1024.0	
rubber granulate (S850)	S850_01	840.5	865.25
	S850_02	848.0	
	S850_03	870.5	
	S850_04	902.0	
rubber granulate with rubber fibers (F570)	F570_01	606.5	622.5
	F570_02	621.5	
	F570_03	626.0	
	F570_04	636.0	
rubber granulate with rubber fibers (F700)	F700_01	697.5	719.5
	F700_02	709.5	
	F700_03	723.5	
	F700_04	747.5	
rebound polyurethane (P150)	P150_01	142.0	146.75
	P150_02	144.0	
	P150_03	150.0	
	P150_04	151.0	
rebound polyurethane (P250)	P250_01	243.0	257.25
	P250_02	254.0	
	P250_03	262.0	
	P250_04	270.0	

**Table 2:** Parameters of machine used for dynamic stiffness test.

Device Name/Manufacturer	Key Feature	Key Value of Parameters
Dynamic exciter—Brüel & Kjær (Virum, Denmark) Mini-shaker Type 4810	Provides sinusoidal force	Sine peak max 10 N Frequency range DC-18 kHz
IEPE accelerometer—MMF (Radebeul, Germany) KS78B.100	Measures acceleration of system response	Peak acceleration 60 g (~600 m/s^2^) Linear frequency range (5% deviation) 0.6 Hz–14 kHz
Force sensor—Forsentek (Shenzhen, China) FSSM 50 N	Measures force applied to system	Max force 50 N Rated output 2.0 mV/V Hysteresis ± 0.1% R.O. (rated output)
Dynamic stiffness test bench	Measures resonant frequency of sample (200 mm × 200 mm × 50 mm) under load of 8 kg	Linear frequency range upper limit (5% deviation) 20–250 Hz—measured

**Table 3:** Results showing the critical damping factor, resonant frequency, and dynamic stiffness of the tested samples obtained from the reference method.

Material Name	CDF (−) (95%CI)	Rf (Hz) (95%CI)	DS (MN/m^3^) (95%CI)
rubber granulate with rubber fibers (F570)	0.0675	73.28	42.4
	(0.0581, 0.0769)	(69.24, 77.31)	(37.9, 47)
rubber granulate with rubber fibers (F700)	0.0674	77.61	47.6
	(0.064, 0.0709)	(74.32, 80.9)	(43.6, 51.6)
rebound polyurethane (P150)	0.0691	35.5	10.0
	(0.0501, 0.0881)	(33.8, 37.2)	(9.0, 10.9)
rebound polyurethane (P250)	0.0805	69.97	38.7
	(0.0699, 0.0911)	(64.93, 75.02)	(33.1, 44.3)
rubber granulate (S1000)	0.1127	76.31	46.1
	(0.1023, 0.1231)	(70.18, 82.43)	(38.7, 53.4)
rubber granulate (S850)	0.1123	84.39	56.3
	(0.0962, 0.1284)	(78.88, 89.89)	(49, 63.6)

**Table 4:** Results showing relative bounce height values obtained from ball tracking.

	1st Peak/Start (95%CI)	2nd/1st Peak (95%CI)	3rd/2nd Peak (95%CI)	4th/3rd Peak (95%CI)
rubber granulate with rubber fibers (F570)	0.4877	0.5134	0.5091	0.497
	(0.4703, 0.5052)	(0.4995, 0.5272)	(0.4985, 0.5197)	(0.4845, 0.5095)
rubber granulate with rubber fibers (F700)	0.5183	0.5549	0.5595	0.5547
	(0.5027, 0.5338)	(0.5356, 0.5743)	(0.5485, 0.5705)	(0.5455, 0.564)
rebound polyurethane (P150)	0.4764	0.4973	0.4819	0.4455
	(0.4625, 0.4903)	(0.4893, 0.5053)	(0.4623, 0.5014)	(0.4271, 0.4639)
rebound polyurethane (P250)	0.4935	0.5199	0.5158	0.4981
	(0.4871, 0.4999)	(0.5103, 0.5295)	(0.5063, 0.5253)	(0.4845, 0.5116)
rubber granulate (S1000)	0.3701	0.3763	0.3555	0.363
	(0.3353, 0.405)	(0.3366, 0.4159)	(0.3299, 0.3812)	(0.3131, 0.4128)
rubber granulate (S850)	0.3957	0.4183	0.4114	0.3902
	(0.3259, 0.4654)	(0.3648, 0.4718)	(0.3703, 0.4525)	(0.329, 0.4513)

**Table 5:** Results showing relative indentation values obtained from tracking the ball’s path.

	1st Ind. */Start (95%CI)	2nd/1st Ind. (95%CI)	3rd/2nd Ind. (95%CI)	4th/3rd Ind. (95%CI)
rubber granulate with rubber fibers (F570)	0.0201	0.0281	0.0373	0.0451
	(0.0187, 0.0215)	(0.0257, 0.0304)	(0.0335, 0.0412)	(0.0406, 0.0496)
rubber granulate with rubber fibers (F700)	0.0177	0.0263	0.0323	0.0382
	(0.0167, 0.0187)	(0.0223, 0.0303)	(0.0241, 0.0404)	(0.027, 0.0494)
rebound polyurethane (P150)	0.0547	0.0825	0.1132	0.1375
	(0.0505, 0.0589)	(0.0778, 0.0872)	(0.1002, 0.1263)	(0.1218, 0.1533)
rebound polyurethane (P250)	0.0293	0.0415	0.0547	0.0675
	(0.0252, 0.0335)	(0.036, 0.0469)	(0.0453, 0.064)	(0.0567, 0.0782)
rubber granulate (S1000)	0.0084	0.0172	0.0269	0.0348
	(0.0064, 0.0104)	(0.0136, 0.0208)	(0.0205, 0.0333)	(0.0229, 0.0467)
rubber granulate (S850)	0.013	0.0245	0.0378	0.0479
	(0.0108, 0.0152)	(0.0161, 0.0329)	(0.0249, 0.0507)	(0.0324, 0.0634)

* Ind. means indentation.

**Table 6:** Initial goodness of fit results for val(x) = p1·x + p2 function, where x is relative indentation and val(x) is dynamic stiffness.

	1st Ind./Start	2nd/1st Ind.	3rd/2nd Ind.	4th/3rd Ind.	5th/4th Ind.
SSE	747.6245	788.0932	911.2817	969.5523	1059.64
R^2^	0.858681	0.851032	0.827746	0.816732	0.799703
RMSE	5.829488	5.985183	6.435984	6.638566	6.940134

**Table 7:** Initial goodness of fit results for val(x) = p1·x + p2 function, where x is relative rebound and y is critical damping factor.

	1st Peak/Start	2nd/1st Peak	3rd/2nd Peak	4th/3rd Peak
SSE	0.001982	0.002138	0.002646	0.003137
R^2^	0.814922	0.800411	0.752947	0.707063
RMSE	0.009492	0.009857	0.010967	0.011942

## Data Availability

Data are contained within the article.
